# Characterization of Clinical Symptoms by Race Among Women With Early-Stage, Hormone Receptor–Positive Breast Cancer Before Starting Chemotherapy

**DOI:** 10.1001/jamanetworkopen.2021.12076

**Published:** 2021-06-01

**Authors:** Xin Hu, Puneet K. Chehal, Cameron Kaplan, Rebecca A. Krukowski, Roy H. Lan, Edward Stepanski, Lee Schwartzberg, Gregory Vidal, Ilana Graetz

**Affiliations:** 1 Department of Health Policy and Management, Rollins School of Public Health, Emory University, Atlanta, Georgia; 2 Gehr Family Center for Health Systems Science and Innovation, Keck School of Medicine of University of Southern California, Los Angeles; 3 College of Medicine, University of Tennessee Health Science Center, Memphis; 4 ConcertAI, Boston, Massachusetts; 5 West Cancer Center and Research Institute, Germantown, Tennessee; 6 Division of Hematology and Oncology, University of Tennessee Health Science Center, Memphis

## Abstract

**Importance:**

Race disparities persist in breast cancer mortality rates. One factor associated with these disparities may be differences in symptom burden, which may reduce chemotherapy tolerance and increase early treatment discontinuation.

**Objectives:**

To compare symptom burden by race among women with early-stage breast cancer before starting chemotherapy and quantify symptom differences explained by baseline characteristics.

**Design, Setting, and Participants:**

A cross-sectional analysis of symptom burden differences by race among Black and White women with a diagnosis of stage I to III, hormone receptor–positive breast cancer who had a symptom report collected before chemotherapy initiation in a large cancer center in the southern region of the US from January 1, 2007, through December 31, 2015. Analyses were conducted from November 1, 2019, to March 31, 2021. Blinder-Oaxaca decomposition was used, adjusting for baseline sociodemographic and clinical characteristics.

**Main Outcomes and Measures:**

Four symptom composite scores with a mean (SD) of 50 (10) were reported before starting chemotherapy (baseline) and were derived from symptom items: general physical symptoms (11 items), treatment adverse effects (8 items), acute distress (4 items), and despair (7 items). Patients rated the severity of each symptom they experienced in the past week on a scale of 0 to 10 (where 0 indicates not a problem and 10 indicates as bad as possible).

**Results:**

A total of 1338 women (mean [SD] age, 54.6 [11.6] years; 420 Black women [31.4%] and 918 White women [68.6%]) were included in the study. Before starting chemotherapy, Black women reported a statistically significantly higher (ie, worse) symptom composite score than White women for adverse effects (44.5 vs 43.8) but a lower acute distress score (48.5 vs 51.0). Decomposition analyses showed that Black patients’ characteristics were associated with higher symptom burden across all 4 scores. However, these differences were offset by relatively greater, statistically significant, unexplained physical, distress, and despair symptom reporting by White patients.

**Conclusions and Relevance:**

In this study, before starting chemotherapy, Black patients with early-stage breast cancer reported significantly higher burden for symptoms that may be exacerbated with chemotherapy and lower distress symptoms compared with White patients. Future studies should explore how symptoms change before and after treatment and differ by racial/ethnic groups and how they are associated with treatment adherence and mortality disparities.

## Key Points

**Question:**

Are there symptom burden differences between Black and White women with early-stage breast cancer before starting chemotherapy?

**Findings:**

In this cross-sectional observatonal study of 1338 patients from 1 cancer center, Black women reported a higher burden for symptoms typically associated with chemotherapy and lower distress than White women before chemotherapy intiation. Black patients’ baseline characteristics were associated with a significantly higher symptom burden, but this symptom burden was offset by relatively greater, unexplained reporting of physical, distress, and despair symptoms by White patients.

**Meaning:**

Capturing treatment-related toxic effects by considering pretreatment symptoms may result in better-informed treatment decisions for patients.

## Teaser

This cohort study compares symptom burden among Black and White women with early-stage breast cancer before starting chemotherapy and assesses symptom differences associated with baseline characteristics.

## Introduction

Although the breast cancer mortality rate has decreased, large racial/ethnic disparities persist.^1,2^ From 2012 to 2016, the breast cancer mortality rate among Black women was 41% higher than among White women.^3^ Although differences in treatment, health care access, and comorbidities explain some of the differences in mortality,^4,5,6,7^ they do not fully explain this disparity. Another important factor may be symptom burden. Severe or uncontrolled physical symptoms and mental health issues may decrease tolerance for the full chemotherapy course and are associated with early treatment discontinuation, which may increase breast cancer mortality.^8,9,10^

Chemotherapy is a critical treatment component for early-stage breast cancer. Compared with White women, Black women are more likely to delay initiation of chemotherapy or discontinue chemotherapy early.^8,11,12,13,14^ Several studies compared symptoms during or after chemotherapy by race/ethnicity and found mixed results.^12,15,16,17,18,19,20,21,22,23,24,25,26,27,28,29,30,31,32,33,34,35,36^ Symptom assessment timing varied in these studies (eg, during treatment vs later in survivorship), which may explain the inconsistent findings. To our knowledge, no studies have compared differences in patient-reported symptoms before starting chemotherapy.

Several studies found that symptom severity differences between Black and White patients were attenuated but remained statistically significant after adjusting for patient characteristics (eg, sociodemographic characteristics, cancer stage, treatment received, and comorbidities).^17,37^ However, it is unclear whether symptom differences were associated with the treatment itself or with preexisting symptom burden not associated with the treatment.^38,39^ By comparing symptoms before treatment initiation, we can establish baseline treatment-related symptom differences between Black and White patients, which is an important step in determining symptom changes associated with treatment.

This study compared patient-reported symptoms before initiating chemotherapy among Black and White women with breast cancer. We used a Blinder-Oaxaca decomposition model to isolate the difference in symptom burden associated differences in baseline characteristics (ie, sociodemographic and clinical characteristics) from the unexplained difference in symptom reporting by Black and White women. We hypothesized that Black patients would report a higher baseline symptom burden than White patients and that most of the differences by race would be explained by associations with baseline characteristics.

## Methods

### Data and Analytical Sample

Our cohort consisted of patients treated at a large multidisciplinary cancer center, the West Cancer Center and Research Institute (WCCRI). At the WCCRI, patient-reported outcomes are routinely collected during visits using the Patient Care Monitor (PCM; ConcertAI). Responses are saved in patients’ electronic health records, and severe symptoms are highlighted for further evaluation during their visit. The WCCRI serves 60% of all oncology patients in the tristate area of west Tennessee, north Mississippi, and east Arkansas. Although the disparity in breast cancer mortality rates between Black and White patients improved in this region during the last 20 years, the mortality rate remains 1.7 times higher for Black women.^5,40^ The University of Tennessee Health Science Center institutional review board approved the study protocol and waived the requirement for written informed consent for participants in this data-only study. We followed the Strengthening the Reporting of Observational Studies in Epidemiology (STROBE) reporting guideline for cross-sectional studies.

We used electronic health record data to identify patients’ diagnosis date, diagnosis stage, and demographic information. We merged electronic health record and PCM data with data from the Tennessee cancer registry to retrieve patients’ cancer history and cancer treatments received outside the WCCRI system. Where available, we obtained enrollment and claim information from Medicare and Tennessee’s Medicaid programs, to identify additional health care services used and chronic conditions.

We included patients who received a diagnosis during the period from January 1, 2007, to December 31, 2015, of a first primary and stage I to III hormone receptor–positive breast cancer at the WCCRI (n = 5807), and we restricted the sample to those who initiated chemotherapy within 180 days of diagnosis (n = 1865). We then identified patients with a PCM report up to 45 days before chemotherapy initiation (n = 1526) and selected the report closest to the initiation date. We further restricted our sample to those who identified as White or Black (n = 1477), given a limited sample of other races/ethnicities, and those with a nonmissing residence zip code (n = 1338) (eFigure in the Supplement).

Comorbidity and surgery are important confounders for symptoms. Therefore, we tested the sensitivity of our findings using the following 2 subset samples: (1) Medicare beneficiaries with continuous fee-for-service coverage and complete claims for comorbidity evaluation in the year before their diagnosis (n = 147) and (2) patients with surgery identified prior to chemotherapy and a PCM report collected between surgery and chemotherapy initiation (n = 658).

### Patient-Reported Symptoms

Our primary outcomes are 4 validated PCM scores derived from symptom items: general physical symptoms (11 items), treatment adverse effects (8 items), acute distress (4 items), and despair (7 items) (eTable 1 in the Supplement).^41^ Patients rated the severity of each symptom they experienced in the past week on a scale of 0 to 10 (where 0 indicates not a problem and 10 indicates as bad as possible). Given low symptom prevalence, we calculated the percentage of patients reporting any severity (0 vs 1-10) for each symptom. Each PCM score was calculated by summing raw item scores and then transformed into a normalized T score with a mean (SD) of 50 (10). Although common physical symptoms are captured in both general physical symptoms and treatment adverse effects scores, the items included in the treatment adverse effects score capture symptoms that may be exacerbated by chemotherapy. Because we included symptoms reported before starting chemotherapy, they should not be interpreted as symptoms caused by chemotherapy. General physical symptoms and treatment adverse effects scores together capture patients’ full profile of physical symptoms.

### Covariates

Patient baseline characteristics included sociodemographic (eg, race, age, neighborhood-level income, educational level, and state of residence) and clinical factors (eg, cancer stage at diagnosis, time between cancer diagnosis and chemotherapy initiation, and time between PCM survey and chemotherapy initiation). Time between cancer diagnosis and chemotherapy was included given evidence that Black women delay initiation of cancer treatment, which may affect outcomes.^11,42,43^ We controlled for the lag between PCM date and chemotherapy initiation because of its considerable variation. Data on neighborhood-level household median income and percentage of adults with a high school degree from the 2011-2015 American Community Survey 5-year estimates file^44^ were linked using patients’ residence zip codes or county codes where zip code–level data were not available.

For subsample analyses of Medicare beneficiaries, we obtained comorbidity data from the Chronic Conditions Segment of Medicare Master Beneficiary Summary File. Twelve common comorbidities were selected according to the National Cancer Institute Comorbidity Index.^45^ Subgroup analyses among patients with prechemotherapy surgery also controlled for time between surgery and the PCM survey.

### Statistical Analysis

Analyses were conducted from November 1, 2019, to March 31, 2021. We used the Blinder-Oaxaca decomposition model to quantify net differences in reported symptoms explained by differences in observable covariates between 2 groups and unexplained differences in symptoms.^46,47,48,49^ Specifically, we used a 2-fold–type decomposition with White patients as the reference group. *Net differences* refer to differences in raw symptom scores between White and Black patients. The explained difference was calculated as the sum of the difference in White and Black patients for each observed characteristic multiplied by the corresponding coefficient from the ordinary linear square model of White patients. A negative explained difference in our outcome variables would mean that, if Black patients had shared the same baseline characteristics as White patients, their symptom burden would be lower (ie, negative). The unexplained difference quantifies the change in Black patients’ symptom scores when applying White patients’ coefficients to Black patients. In our context, we interpreted unexplained differences as differences in reporting patterns between Black and White women not accounted for by covariates in our model.

To explore the extent to which the unexplained differences in symptom scores between Black and White patients were associated with differences in baseline comorbidities or receipt of surgery, we estimated Blinder-Oaxaca decomposition among a subset of Medicare patients with or without comorbidity indicators and a subset of surgery recipients with or without controlling for time from surgery. In the baseline characteristics comparison by race, the *t* test was used for mean comparisons, the Wilcoxon rank sum test was used for median comparisons, and the χ^2^ test or the Fisher exact test were used for categorical measures. All *P* values were from 2-sided tests, and results were deemed statistically significant at *P* < .05. We used SAS software (2002-2012; SAS Institute Inc) for sample creation and Stata (2013; Stata Statistical Software: Release 13; StataCorp LP) for data analysis.

## Results

Among the 1338 women in the study, the mean (SD) age was 54.6 (11.6) years, 918 (68.6%) were White women, and 420 (31.4%) were Black women. Compared with White patients, Black patients were younger (mean [SD], 52.3 [10.9] years vs 55.7 [11.8] years; *P* < .001) and more likely to live in neighborhoods with lower incomes (mean [SD], $42 167 [$18 828] vs $58 091 [$24 704]; *P* < .001) and fewer adults with a high school degree (mean [SD], 81.3% [8.6%] vs 86.2% [9.1%]; *P* < .001) (Table 1).

**Table 1.** Characteristics of Patients

Characteristic	Patients, No. (%)			*P* value^a^
	Total (N = 1338)	Black (n = 420)	White (n = 918)	
Age, y				
Mean (SD)	54.6 (11.6)	52.3 (10.9)	55.7 (11.8)	<.001
Median (IQR)	54.0 (47.0-62.0)	53.0 (45.0-60.0)	55.0 (47.0-64.0)	<.001
Stage at diagnosis				
I	402 (30.0)	115 (27.4)	287 (31.3)	.10
II	679 (50.7)	211 (50.2)	468 (51.0)	
III	257 (19.2)	94 (22.4)	163 (17.8)	
Household income at the neighborhood level, $				
Mean (SD)	53 092 (24 173)	42 167 (18 828)	58 091 (24 704)	<.001
Median (IQR)	50 701 (33 829-65 517)	35 686 (29 193-51 656)	52 180 (36 816-70 541)	<.001
Adults with high school degree at the neighborhood level, %				
Mean (SD)	84.7 (9.3)	81.3 (8.6)	86.2 (9.1)	<.001
Median (IQR)	86.0 (77.0-92.8)	80.0 (75.6-88.4)	87.4 (79.7-94.2)	<.001
State of residence				
Arkansas	58 (4.3)	9 (2.1)	49 (5.3)	.001
Mississippi	367 (27.4)	87 (20.7)	280 (30.5)	
Tennessee	866 (64.7)	319 (76.0)	547 (59.6)	
Other	47 (3.5)	5 (1.2)	42 (4.6)	
Time between survey and chemotherapy initiation, mean (SD), d	5.9 (8.3)	6.1 (9.0)	5.8 (8.0)	.52
Time between diagnosis and chemotherapy initiation, mean (SD), d	60.6 (32.0)	65.7 (35.1)	58.2 (30.2)	<.001
Any severity symptoms, mean (SD), No.	11.8 (9.2)	11.9 (9.3)	11.8 (9.1)	.87

Abbreviation: IQR, interquartile range.

^a^ The *t* test was used for mean comparisons, the Wilcoxon rank-sum test was used for median comparisons, and the χ^2^ test or the Fisher exact test were used for categorical measures.

### General Physical Symptoms

Of 11 symptoms in the general physical symptoms score, patients reported a mean (SD) of 3.2 (2.5) symptoms of any severity. White patients reported 3 symptoms more often than Black patients: fatigue, tiredness, or weakness (597 of 903 [66.1%] vs 219 of 412 [53.2%]; *P* < .001); daytime sleepiness (279 of 917 [30.4%] vs 80 of 416 [19.2%]; *P* < .001); and trouble sleeping (487 of 916 [53.2%] vs 187 of 417 [44.8%]; *P* = .005). Black patients reported sweating more frequently than White patients (153 of 418 [36.6%] vs 257 of 914 [28.1%]; *P* = .002) (Figure).

**Figure.**
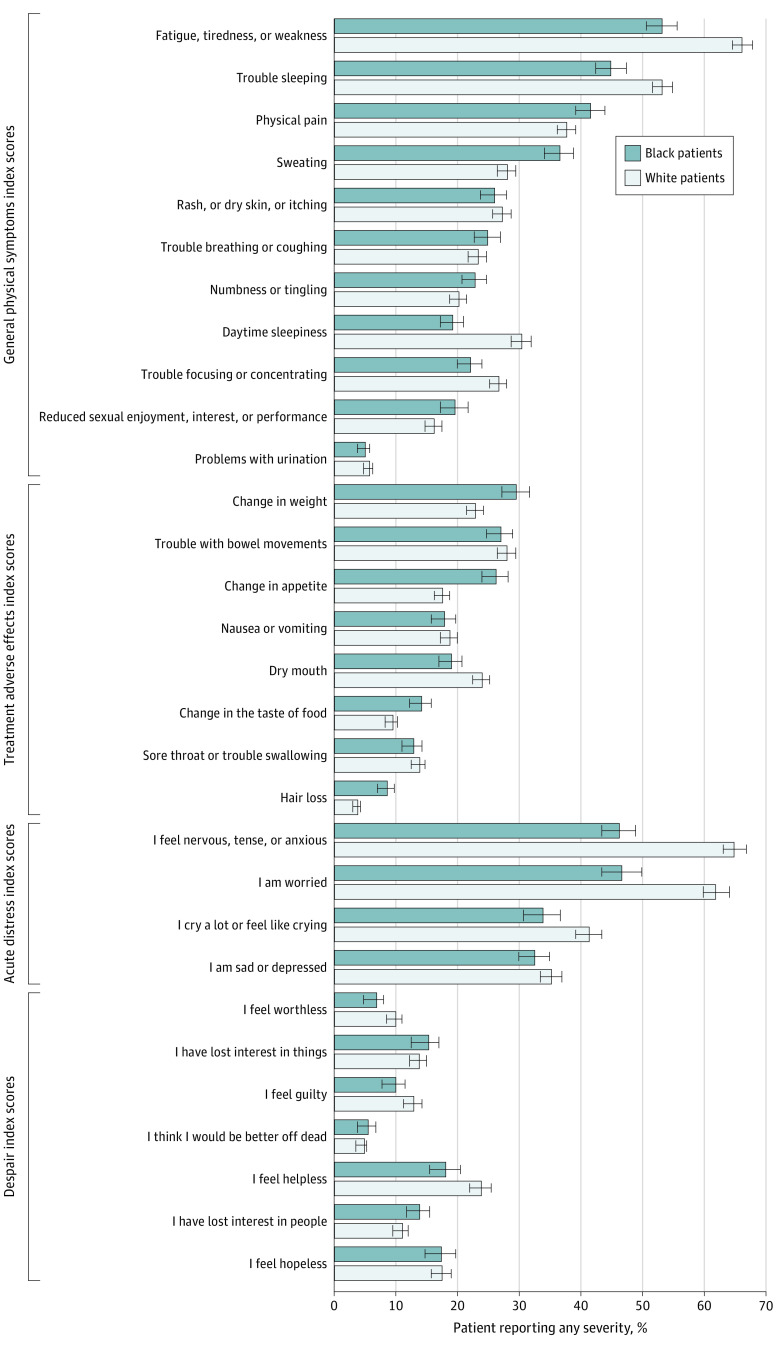
Unadjusted Differences in Reported Symptom Index Scores and Individual Symptoms by Race

White and Black patients reported similar mean (SE) general physical symptom scores (44.7 [0.3] vs 44.5 [0.4]) (Table 2). When the net difference of 0.2 (*P* = .62) was decomposed, we found a negative explained difference. Thus, collectively, Black patient characteristics were associated with 1.1 points higher (ie, worse) general physical symptom scores (*P* < .001). Among the sociodemographic characteristics, neighborhood-level income was associated with the largest proportion of total explained difference (–0.52 of –1.1 [48.3%]) (Table 3). The negative explained difference was offset by the larger, positive unexplained difference; thus, for a given set of characteristics, White patients reported more severe physical symptoms (mean [SE], 1.3 [0.6] points; *P* = .02) (Table 2).

**Table 2.** Blinder-Oaxaca Decomposition of Racial Differences in Symptom Index Scores

Symptom index score	Patients, No.	Unadjusted mean (SE) composite score^a^		Crude net difference (SE) in mean scores^a^	Explained difference (SE)^a^	Unexplained difference (SE)^a^
		White patients	Black patients			
General physical symptoms	1338	44.7 (0.3)^b^	44.5 (0.4)^b^	0.2 (0.5)	−1.1 (0.3)^b^	1.3 (0.6)^b^
Treatment adverse effects	1337	43.8 (0.2)^b^	44.5 (0.3)^b^	−0.7 (0.3)^b^	−0.5 (0.2)^b^	−0.2 (0.3)
Acute distress	1037	51.0 (0.4)^b^	48.5 (0.6)^b^	2.5 (0.8)^b^	−1.2 (0.4)^b^	3.7 (0.8)^b^
Despair	819	48.0 (0.3)^b^	47.6 (0.5)^b^	0.4 (0.6)	−1.1 (0.4)^b^	1.4 (0.7)^b^

^a^ Robust SEs in parentheses. Analysis controlled for age groups, household income at the neighborhood level and adults with high school degree at the neighborhood level, cancer stage, time between cancer diagnosis and chemotherapy, time between survey and chemotherapy, and state of residence.

^b^ *P* < .05.

**Table 3.** Explained Racial Differences in Symptom Index Scores by Each Baseline Characteristic

Characteristic	General physical symptoms		Treatment adverse effects		Acute distress		Despair	
	Explained difference	% of Total explained difference	Explained difference	% of Total explained difference	Explained difference	% of Total explained difference	Explained difference	% of Total explained difference
Total	−1.1^a^	100.0	−0.5^a^	100.0	−1.2^a^	100.0	−1.1^a^	100.0
Age	−0.24^a^	22.5	0.01	−3.0	−0.30^a^	25.8	−0.17	16.4
Disease stage	−0.02	2.0	−0.02	4.9	0.02	−1.9	−0.05	5.1
Household income at the neighborhood level	−0.52^a^	48.3	−0.20	43.1	−0.81^a^	68.9	−0.98^a^	93.2
Adults with high school degree at the neighborhood level	−0.06	5.3	−0.10	21.9	0.02	−1.4	0.37	−35.2
State of residency	−0.06	6.0	−0.05	9.9	−0.32	27.1	−0.13	12.4
Time between survey and chemotherapy initiation	−0.02	14.3	−0.02	19.3	−0.02	−20.0	−0.02	6.2
Time between diagnosis and chemotherapy initiation	−0.15^a^	1.6	−0.09	3.9	0.24	1.3	−0.07	1.9

^a^ *P* < .05.

### Treatment Adverse Effects

With the 8-item treatment adverse effects score, patients reported a mean (SD) of 1.4 (1.7) symptoms with any severity. Most of these symptoms were less common among White patients than Black patients, including weight change (206 of 899 [22.9%] vs 121 of 410 [29.5%]; *P* = .01), appetite change (161 of 916 [17.6%] vs 110 of 419 [26.3%]; *P* < .001), taste changes (86 of 914 [9.4%] vs 59 of 416 [14.2%]; *P* = .01), and hair loss (35 of 915 [3.8%] vs 36 of 418 [8.6%]; *P* < .001) (Figure).

White patients reported lower mean (SE) treatment adverse effects scores than Black patients (43.8 [0.2] vs 44.5 [0.3]; net difference, −0.7; *P* = .02). The explained difference was negative, implying that, if the average Black patient had the same characteristics as the average White patient, she would have reported a 0.5-point lower mean treatment adverse effects score (*P* = .005). Neighborhood-level income and educational level were associated with 43.1% (–0.20 of –0.5) and 21.9% (–0.10 of –0.5) of total explained differences, respectively (Table 3). The remaining unexplained difference was small and not statistically significant (−0.2; *P* = .54) (Table 2).

### Acute Distress

Patients reported a mean (SD) of 1.7 (1.5) of 4 acute distress symptoms of any severity. White patients reported 3 distress symptoms more frequently than Black patients: feeling nervous, tense, or anxious (457 of 705 [64.8%] vs 153 of 331 [46.2%]; *P* < .001), feeling worried (350 of 566 [61.8%] vs 118 of 253 [46.6%]; *P* < .001), and crying a lot or feeling like crying (234 of 566 [41.3%] vs 86 of 254 [33.9%]; *P* = .04) (Figure).

White patients reported significantly higher mean (SE) acute distress scores than Black patients (51.0 [0.4] vs 48.5 [0.6]; net difference, 2.5; *P* = .001) (Table 2). The explained difference was negative, implying that Black patient characteristics were associated with, on average, a 1.2-point higher score (*P* = .005). Most of the explained difference was associated with neighborhood-level income (–0.81 of –1.2 [68.9%]) (Table 3). Higher scores assocated with Black patient characteristics were offset by a relatively larger and positive unexplained difference. For a given set of characteristics, White patients reported a mean (SE) 3.7 (0.8) points higher acute distress score than Black patients (*P* < .001) (Table 2).

### Despair

Patients reported, on average, less than 1 of 7 despair symptoms (mean [SD] score, 0.7 [1.5]) (Figure). Comparisons of individual despair symptoms did not show a statistically significant difference between White and Black patients.

White patients reported a mean (SE) despair score of 48.0 (0.3), and Black patients reported a mean (SE) despair score of 47.6 (0.5) (net difference, 0.4; *P* = .55) (Table 2). The decomposition model found an explained difference of −1.1 points (*P* = .003), which was mostly associated with neighborhood-level income (–0.98 of –1.1 [93.2%] of total explained difference [Table 3]). Thus, if a Black patient had the same characteristics as a White patient, she would have reported a 1.1-point lower (or better) despair score. The explained difference was offset by a relatively larger positive mean (SE) unexplained difference of 1.4 (0.7) points (*P* = .049), resulting in a positive crude difference in mean despair score (Table 2).

### Sensitivity Analyses

Among the 147 Medicare beneficiaries, we compared Blinder-Oaxaca decomposition models with or without adjusting for comorbidities. We found that, by adjusting for comorbidities, the explained differences were larger and statistically significant in all 4 scores. Nevertheless, it did not change the overall direction of results, which were comparable with the main cohort results (eTable 2 and eTable 4 in the Supplement). Analyses among 658 patients with a documented prechemotherapy surgery date with or without adjusting for time from surgery showed similar results and were consistent with main analyses as well. This finding suggests the minimal confoundedness of surgery on the baseline chemotherapy symptom report (eTable 3 and eTable 5 in the Supplement).

## Discussion

To our knowledge, this is the first study to compare patient-reported physical and psychological symptoms among Black and White women with breast cancer before starting chemotherapy. As expected, symptom burden was considerably lower than in previous studies in which assessments were taken after chemotherapy started.^16,17,18,23,25^ We found race-based differences in reported symptoms, with White women reporting more severe distress symptoms and Black women reporting more severe symptoms typically associated with chemotherapy treatment.

For all composite scores, decomposition of differences between Black and White patients consistently showed a negative and statistically significant explained difference. Thus, if Black patients had the same characteristics as White patients, such as living in neighborhoods with higher incomes, they would have reported a lower symptom burden. Neighborhood-level income had the most explanatory power (43.1%-93.2% of total explained difference) among the included baseline characteristics. Moreover, the positive and statistically significant unexplained difference found in physical, distress, and despair symptom scores suggests that White women reported significantly more severe symptoms than Black women with comparable characteristics. In other words, given Black patients’ characteristics, if they reported symptoms as if they were the average White patient, Black patients’ symptom burden scores would be higher (ie, worse). This pattern provides evidence of race-based differences in symptom reporting and illustrates the insights gained from exploring the masked underlying variations in symptom reporting by race.

It is concerning that symptoms that may be exacerbated with chemotherapy were more severe at baseline among Black patients.^8,9,10^ Among specific symptoms, Black women were more likely to report appetite changes, weight changes, changes in taste, and hair loss before starting chemotherapy. The Blinder-Oaxaca decomposition found that a large proportion (71%, −0.5 explained of −0.7 net difference) of the difference between Black and White patients in baseline adverse effects was associated with the difference in observed patient characteristics. Unlike the other composite scores, we did not find evidence of unexplained higher reporting of treatment adverse effects among White women. Having higher baseline symptoms that may be exacerbated with chemotherapy may be associated with even higher symptom burden during treatment, potentially contributing to the greater treatment discontinuation rates observed among Black women.^50^ Without accounting for baseline symptoms, treating oncologists may incorrectly assume that these symptoms are associated with chemotherapy itself rather than patients’ characteristics. This assumption may result in treatment course changes due to misattributed chemotherapy-related toxic effects. Therefore, oncologists should consider making treatment decisions based on changes in treatment-related symptoms reported before and after chemotherapy initiation.

Our finding that White women reported greater distress than Black women is consistent with previous epidemiologic surveys that Black women report lower levels of distress compared with White women during cancer treatment and in later survivorship.^51,52,53^ Prior research identified several potential protective factors that may be associated with this difference (eg, resilience, social support, and religious beliefs).^51,54^ Moreover, Black women may use different coping strategies (eg, suppressing emotions and wishful thinking) that may be associated with lower reporting of distress, whereas White women may be more comfortable expressing emotions.^55^ Accordingly, our decomposition results suggest that, if Black women had the same characteristics as White women, they would have reported even lower (ie, better) distress composite scores, widening the gap between the 2 groups. More research is needed to investigate the causes of this racial/ethnic difference in distress.

Across all 4 composite scores, neighborhood-level income was most associated with the observed differences in symptoms by race, ranging from 43.1% of the difference in treatment adverse effects to 93.2% for distress. Thus, oncologists, hospitals, and related public health authorities should consider the social context when evaluating the source of racial differences in symptoms and subsequent cancer outcomes. Targeted social support programs for patients living in underresourced communities may be beneficial.

### Limitations

Our study has several limitations. Unexplained differences in reported symptoms may have been associated with both differences in actual prevalence of symptoms and systemic differences in reporting between the 2 groups. For example, although it is possible that Black women truly experienced less fatigue and had lower distress and despair than White patients, these differences may also be partially explained by interpretation of the questions and reporting preferences.^56^ It is also possible that Black patients underreported symptoms owing to fears that racial bias may interfere with their physician’s decision about their treatment.^57^ More research is needed to fully understand the reporting process, including how patient perceptions and patient-clinician interactions are associated with treatment strategies and decision-making. Measurement equivalence for different racial/ethnic groups is a concern for patient-reported outcome assessment,^58,59,60^ and we cannot separate out such an effect given the study data. In addition, interpretation of the explained and unexplained differences depends on the availability of observable characteristics. Although our sensitivity analyses addressed the potential confoundedness of comorbidities and symptoms from surgery, there may be other potential confounders, such as surgery types, other treatments, lifestyle factors, and social support, that we were not able to measure and include in our models.^61^ Furthermore, we studied women with hormone receptor–positive early-stage breast cancer who were treated in a single cancer center in the southern US. Therefore, our results may not be generalizable to other conditions or settings.

## Conclusions

In this large, single-center cross-sectional study of 1338 women with a diagnosis of early-stage breast cancer between 2007 and 2015, we found 2 key differences in symptoms before chemotherapy among Black and White women. First, if Black patients had the same baseline characteristics as White patients, they would have had a lower symptom burden across all 4 symptom scores. However, this symptom burden was not always observable when comparing crude differences in average symptoms because it was offset by larger, statistically significant, unexplained reporting of more severe symptoms by White patients. Second, in contrast to other symptom measures, the mean treatment adverse effects score before starting chemotherapy was higher among Black patients. Higher baseline symptoms may be associated with even higher reported treatment adverse effects during chemotherapy. Consequently, capturing treatment-related toxic effects by comparing changes from pretreatment symptoms may result in better-informed treatment decisions for patients. Future studies should examine how prechemotherapy symptom differences by race/ethnicity may be associated with differences in symptom changes during chemotherapy and their subsequent association with treatment adherence and cancer survival.

## Notes

Supplement. **eFigure.** Sample Selection Flow Chart **eTable 1.** List of Individual Symptoms Used for Four Compound Scores **eTable 2.** Patient Characteristics of Medicare Subsample **eTable 3.** Patient Characteristics of Patients Who Received Surgery Before Chemotherapy and Had a PCM Report Between Surgery and Chemotherapy Initiation **eTable 4.** Oaxaca Decomposition of Race Differences in Symptom Index Scores of Medicare Subsample **eTable 5.** Oaxaca Decomposition of Race Differences in Symptom Index Scores of Patients Who Received Surgery Before Chemotherapy and Had a PCM Report Between Surgery and Chemotherapy Initiation
