# Supplementary material for: Characterization of Clinical Symptoms by Race Among Women With Early-Stage, Hormone Receptor–Positive Breast Cancer Before Starting Chemotherapy
Source: JAMA Netw Open. 2021 Jun 1;4(6):e2112076. doi: 10.1001/jamanetworkopen.2021.12076 (PMC8170541; doi:10.1001/jamanetworkopen.2021.12076)
Supplement: Supplement. — eFigure. Sample Selection Flow Chart eTable 1. List of Individual Symptoms Used for Four Compound Scores eTable 2. Patient Characteristics of Medicare Subsample eTable 3. Patient Characteristics of Patients Who Received Surgery Before Chemotherapy and Had a PCM Report Between Surgery and Chemotherapy Initiation eTable 4. Oaxaca Decomposition of Race Differences in Symptom Index Scores of Medicare Subsample eTable 5. Oaxaca Decomposition of Race Differences in Symptom Index Scores of Patients Who Received Surgery Before Chemotherapy and Had a PCM Report Between Surgery and Chemotherapy Initiation [file jamanetwopen-e2112076-s001.pdf]

Supplementary Online Content

Hu X, Chehal PK, Kaplan C, et al. Characterization of clinical symptoms by race among women with early-stage, hormone receptor–positive breast cancer before starting chemotherapy. *JAMA Netw Open.* 2021;4(6):e2112076.
doi:10.1001/jamanetworkopen.2021.12076

eFigure. Sample Selection Flow Chart

eTable 1. List of Individual Symptoms Used for Four Compound Scores

eTable 2. Patient Characteristics of Medicare Subsample

eTable 3. Patient Characteristics of Patients Who Received Surgery Before Chemotherapy and Had a PCM Report Between Surgery and Chemotherapy Initiation

eTable 4. Oaxaca Decomposition of Race Differences in Symptom Index Scores of Medicare Subsample

eTable 5. Oaxaca Decomposition of Race Differences in Symptom Index Scores of Patients Who Received Surgery Before Chemotherapy and Had a PCM Report Between Surgery and Chemotherapy Initiation

This supplementary material has been provided by the authors to give readers additional information about their work.

© 2021 Hu X et al. *JAMA Network Open.*

eFigure. Sample Selection Flow Chart

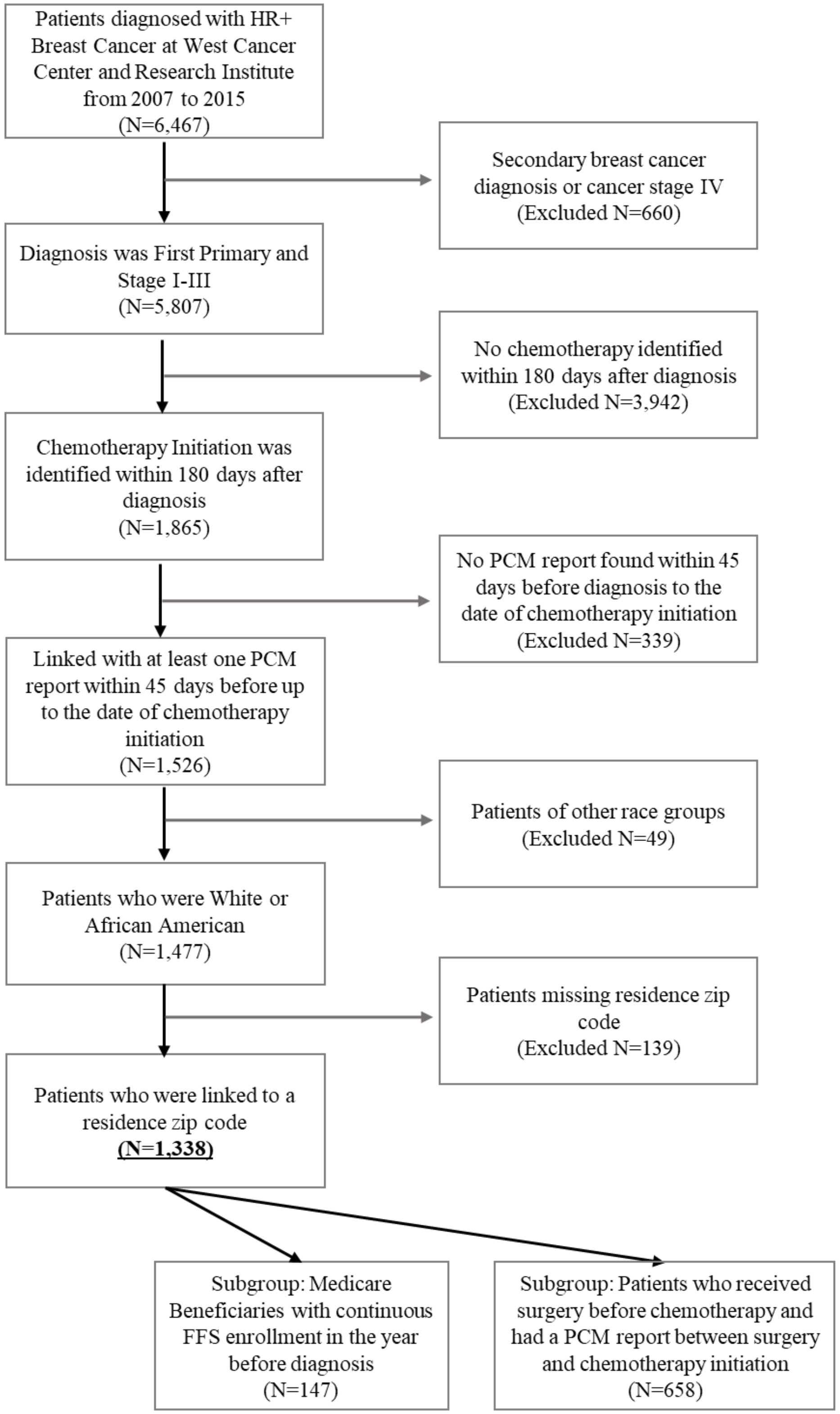

© 2021 Hu X et al. *JAMA Network Open.*

eTable 1. List of Individual Symptoms Used for Four Compound Scores

**Compound Score**	General Physical Symptoms	Treatment Adverse Effects	Acute Distress	Despair
**Individual Item**	1. Fatigue, tiredness, or weakness	1. Change in weight	1. I cry a lot or feel like crying	1. I feel hopeless
	2. Rash or dry skin or itching	2. Hair loss	2. I am worried	2. I have lost interest in people that I used to want to be around
	3. Trouble focusing or concentrating	3. Nausea or vomiting	3. I feel nervous, tense, or anxious	3. I feel helpless
	4. Daytime sleepiness	4. Dry mouth	4. I am sad or depressed	4. I think I would be better off dead
	5. Physical pain	5. Change in the taste of food		5. I feel guilty
	6. Sweating	6. Trouble with bowel movements (diarrhea or constipation)		6. I have lost interest in things that I used to want to do
	7. Trouble breathing or coughing	7. Sore throat or trouble swallowing		7. I feel worthless
	8. Reduced sexual enjoyment, interest, or performance	8. Change in appetite		
	9. Trouble sleeping			
	10. Problems with urination			
	11. Numbness or tingling			

© 2021 Hu X et al. *JAMA Network Open.*

eTable 2. Patient Characteristics of Medicare Subsample

Characteristics	Patients, No. (%)			*P* Value[a]
	Total (N=147)	Black (n=32)	White (n=115)	
Age, y				
Mean (SD)	70.8 (8.5)	65.3 (11.1)	72.3 (6.9)	0.001
Median (IQR)	71.0 (69.0- 76.0)	69.0 (57.5- 72.5)	72.0 (69.0- 77.0)	
Stage at diagnosis				0.81
Stage I	40 (27.2%)	9 (28.1%)	31 (27.0%)	
Stage II	67 (45.6%)	13 (40.6%)	54 (47.0%)	
Stage III	40 (27.2%)	10 (31.3%)	30 (26.1%)	
Comorbidities				0.26
No comorbidity	56 (38.1%)	11 (34.4%)	45 (39.1%)	
One comorbidity	44 (29.9%)	7 (21.9%)	37 (32.2%)	
More than 1 comorbidity	47 (32.0%)	14 (43.8%)	33 (28.7%)	
Household income at the neighborhood level, $				
Mean (SD)	48643 (23271)	31170 (14984)	53505 (22868)	<.001
Median (IQR)	40221 (32531-64209)	29193 (25989-34128)	50701 (34072-64582)	<.001
Adults with high school degree at the neighborhood level, %				
Mean (SD)	82.5 (9.1)	74.6 (7.0)	84.7 (8.4)	<.001
Median (IQR)	82.7 (75.6- 90.7)	75.6 (70.6- 76.8)	86.0 (77.8- 91.5)	<.001
State of residence				0.61
Arkansas	7 (4.8%)	1 (3.1%)	6 (5.2%)	
Mississippi	55 (37.4%)	15 (46.9%)	40 (34.8%)	
Tennessee	82 (55.8%)	16 (50.0%)	66 (57.4%)	
Other	3 (2.0%)	0 (0.0%)	3 (2.6%)	
Time between survey and chemotherapy initiation, mean (SD), d	6.7 (9.9)	5.6 (8.9)	7.0 (10.2)	0.51
Time between diagnosis and chemotherapy initiation, mean (SD), d	69.2 (34.3)	65.3 (41.7)	70.3 (32.1)	0.47
Any Severity Symptoms, mean (SD), No.	12.5 (10.0)	12.4 (10.8)	12.5 (9.8)	0.97

Abbreviation: IQR=Interquartile Range; SD=Standard Deviation

[a] The t test was used for mean comparisons, the Wilcoxon rank-sum test was used for median comparisons, and the χ^2 test or the Fisher exact test 2 were used for categorical measures

eTable 3. Patient Characteristics of Patients Who Received Surgery Before Chemotherapy and Had a PCM Report Between Surgery and Chemotherapy Initiation

Characteristics	Patients, No. (%)			*P* Value[a]
	Total (N=658)	Black (n=210)	White (n=448)	
Age, y				
Mean (SD)	56.2 (11.9)	53.0 (10.8)	57.7 (12.1)	<.001
Median (IQR)	56.0 (48.0, 65.0)	53.0 (46.0, 61.0)	57.0 (49.0, 67.0)	<.001
Stage at diagnosis				0.075
Stage I	238 (36.2%)	63 (30.0%)	175 (39.1%)	
Stage II	304 (46.2%)	105 (50.0%)	199 (44.4%)	
Stage III	116 (17.6%)	42 (20.0%)	74 (16.5%)	
Household income at the neighborhood level, $				
Mean (SD)	52432 (23790)	41177 (18923)	57708 (24021)	<.001
Median (IQR)	48234 (33295-64985)	34128 (29066-51295)	51656 (36993-70541)	<.001
Adults with high school degree at the neighborhood level, %				
Mean (SD)	84.6 (9.0)	81.0 (8.5)	86.3 (8.7)	<.001
Median (IQR)	86.0 (76.9-92.8)	79.6 (75.6-87.1)	87.2 (79.7-94.3)	<.001
State or residence				<.001
Arkansas	25 (3.8%)	5 (2.4%)	20 (4.5%)	
Mississippi	170 (25.8%)	34 (16.2%)	136 (30.4%)	
Tennessee	446 (67.8%)	170 (81.0%)	276 (61.6%)	
Other	17 (2.6%)	1 (0.5%)	16 (3.6%)	
Time between survey and chemotherapy initiation, Mean (SD), d	5.9 (8.6)	6.0 (8.8)	5.8 (8.5)	0.73
Time between diagnosis and chemotherapy initiation, mean (SD), d	73.7 (28.9)	79.5 (31.4)	71.1 (27.2)	0.001
Time between surgery and survey, mean (SD), d	42.1 (25.5)	45.5 (27.2)	40.5 (24.5)	0.019
Any Severity Symptoms, mean (SD), No.	11.7 (9.3)	12.0 (9.5)	11.6 (9.2)	0.61

Abbreviation: IQR=Interquartile Range; SD=Standard Deviation

[a] The t test was used for mean comparisons, the Wilcoxon rank-sum test was used for median comparisons, and the χ^2 test or the Fisher exact test 2 were used for categorical measures

© 2021 Hu X et al. *JAMA Network Open.*

eTable 4. Oaxaca Decomposition of Race Differences in Symptom Index Scores of Medicare Subsample

Symptom Index Scores	Patients, No.	Unadjusted mean (SE) composite score[a]		Crude net Difference (SE) in mean scores[a]	Explained Difference (SE) [a]	Unexplained Difference (SE) [a]
		White patients	Black patients			
**(A) Without Controlling for Comorbidities**						
General Physical Symptoms	147	44.6 (0.7)[b]	43.7 (1.7)[b]	0.9 (1.8)	-3.3 (1.3)[b]	4.2 (2.2)
Treatment Adverse Effects	146	44.5 (0.5)[b]	45.2 (1.2)[b]	-0.8 (1.3)	-1.0 (0.9)	0.2 (1.6)
Acute Distress	105	49.3 (1.3)[b]	45.7 (2.1)[b]	3.6 (2.5)	-3.3 (2.4)	6.9 (3.5)[b]
Despair	73	47.3 (1.2)[b]	46.5 (1.9)[b]	0.8 (2.2)	-6.1 (2.9)[b]	6.9 (3.7)
**(B) Controlling for Comorbidities**						
General Physical Symptoms	147	44.6 (0.7)[b]	43.7 (1.7)[b]	0.9 (1.9)	-3.8 (1.4)[b]	4.7 (2.2)[b]
Treatment Adverse Effects	146	44.5 (0.5)[b]	45.2 (1.2)[b]	-0.8 (1.3)	-1.4 (1.1)	0.6 (1.7)
Acute Distress	105	49.3 (1.3)[b]	45.7 (2.2)[b]	3.6 (2.5)	-3.3 (2.5)	6.9 (3.6)
Despair	73	47.3 (1.2)[b]	46.5 (2.0)[b]	0.8 (2.3)	-6.4 (3.1)[b]	7.2 (3.9)

[a] Robust SEs in parentheses. Analysis controlled for all covariates in the whole sample analysis as well as comorbidities (in panel B).

[b] $P < 0.05$

© 2021 Hu X et al. *JAMA Network Open.*

eTable 5. Oaxaca Decomposition of Race Differences in Symptom Index Scores of Patients Who Received Surgery Before Chemotherapy and Had a PCM Report Between Surgery and Chemotherapy Initiation

Symptom Index Scores	Patients, No.	Unadjusted mean (SE) composite score[a]		Crude net Difference (SE) in mean scores[a]	Explained Difference (SE) [a]	Unexplained Difference (SE) [a]
		White patients	Black patients			
**(C) Without Controlling for Days from Surgery to Survey**						
General Physical Symptoms	658	44.7 (0.4)[b]	44.5 (0.6)[b]	0.1 (0.7)	-1.6 (0.5)[b]	1.7 (0.9)[b]
Treatment Adverse Effects	658	43.7 (0.2)[b]	44.7 (0.4)[b]	-0.9 (0.4)[b]	-0.6 (0.3)[b]	-0.3 (0.5)
Acute Distress	513	50.9 (0.6)[b]	48.3 (0.9)[b]	2.6 (1.1)[b]	-2.5 (0.7)[b]	5.0 (1.3)[b]
Despair	396	48.4 (0.5)[b]	47.8 (0.7)[b]	0.7 (0.9)	-2.0 (0.6)[b]	2.7 (1.1)[b]
**(D) Controlling for Days from Surgery to Survey**						
General Physical Symptoms	658	44.7 (0.4)[b]	44.5 (0.6)[b]	0.1 (0.7)	-1.6 (0.5)[b]	1.7 (0.9)[b]
Treatment Adverse Effects	658	43.7 (0.2)[b]	44.7 (0.4)[b]	-0.9 (0.4)[b]	-0.6 (0.3)[b]	-0.3 (0.5)
Acute Distress	513	50.9 (0.6)[b]	48.3 (0.9)[b]	2.6 (1.1)[b]	-2.4 (0.7)[b]	5.0 (1.3)[b]
Despair	396	48.4 (0.5)[b]	47.8 (0.8)[b]	0.7 (0.9)	-2.0 (0.6)[b]	2.7 (1.1)[b]

[a] Robust SEs in parentheses. Analysis controlled for all covariates in the whole sample analysis as well as time from surgery to survey (in panel D).

[b] P <0.05

© 2021 Hu X et al. *JAMA Network Open.*
